# *Clostridium difficile* in patients attending tuberculosis hospitals in Cape Town, South Africa, 2014–2015

**DOI:** 10.4102/ajlm.v7i2.846

**Published:** 2018-12-06

**Authors:** Brian R. Kullin, Sharon Reid, Valerie Abratt

**Affiliations:** 1 Department of Molecular and Cell Biology, Faculty of Science, University of Cape Town, Cape Town, South Africa

## Abstract

**Background:**

Diarrhoea due to *Clostridium difficile* infection (CDI) poses a significant burden on healthcare systems around the world. However, there are few reports on the current status of the disease in sub-Saharan Africa.

**Objectives:**

This study examined the occurrence of CDI in a South African population of tuberculosis patients, as well as the molecular epidemiology and antibiotic susceptibility profiles of *C. difficile* strains responsible for disease.

**Methods:**

Toxigenic *C. difficile* in patients with suspected CDI attending two specialist tuberculosis hospitals in the Cape Town area were detected using a PCR-based diagnostic assay (Xpert^®^ *C. difficile*). *C. difficile* strains isolated from PCR-positive specimens were characterised by ribotyping, multilocus variable-number tandem-repeat analysis and antibiotic susceptibility testing.

**Results:**

The period prevalence of CDI was approximately 70.07 cases per 1000 patient admissions. Strains belonging to ribotype 017 (RT017) made up over 95% of the patient isolates and all of them were multi-drug resistant. Multilocus variable-number tandem-repeat analysis revealed several clusters of highly related *C. difficile* RT017 strains present in tuberculosis patients in several wards at each hospital.

**Conclusion:**

Tuberculosis patients represent a population that may be at an increased risk of developing CDI and, in addition, may constitute a multi-drug resistant reservoir of this bacterium. This warrants further investigation and surveillance of the disease in this patient group and other high-risk patient groups in sub-Saharan Africa.

## Introduction

*Clostridium difficile* infection (CDI) is the most common cause of nosocomial diarrhoea in the developed world, with complications of the disease, including potentially life-threatening pseudomembranous colitis and toxic megacolon.^1^ The total costs of CDI treatment are estimated to be as high as $2871.00 (United States dollars) to $4846.00 per patient in the United States and between $5243.00 and $8570.00 per patient in European countries,^2^ and there is evidence to suggest that treatment failure and disease relapse rates are increasing.^3^ Since the turn of the century, several significant outbreaks of CDI have occurred and have been documented in North America and Europe.^4,5,6^

Toxigenic *C. difficile* strains produce two large clostridial toxins, TcdA and TcdB, both of which show cytotoxic activity and are responsible for the majority of disease symptoms.^7^ A third binary toxin, CdtAB is also produced by some strains, such as those belonging to ribotype 027 (RT027) and RT078, and may be associated with increased disease severity in some settings.^8^ Several toxin variant strains have also been identified. The most common of these is a subset of strains that produce only one functional toxin (TcdB) and is mainly comprised of members of the RT017 group. This group is widespread in Asia^9^ and is capable of causing severe disease across diverse populations.^6^

The most common risk factor for the development of CDI is previous exposure to antibiotics, particularly clindamycin and cephalosporins and, less frequently, fluoroquinolones.^10^ The resulting dysbiosis allows proliferation of *C. difficile* and the progression of disease symptoms. Additional risk factors include advanced age,^11^ gastric acid suppression therapy^12^ (although this is sometimes debated^13^), co-morbidities such as HIV^14^ and exposure to long-term healthcare facilities.^15^ There have also been several case reports of CDI occurring in patients with tuberculosis,^16,17,18,19^ possibly related to the long-term intensive antibiotic therapy that they typically receive. However, there have been very few studies to date specifically looking at CDI in this patient group. Chang et al.^20^ examined a potential link between fluoroquinolone use and CDI in tuberculosis patients in Hong Kong and concluded that the risk of CDI was moderate for these patients, while Lee et al.^21^ detected a relatively low incidence of CDI in Korean tuberculosis patients (2.83 cases per 1000 adults). More recently, however, Legenza et al.^22^ identified tuberculosis as an independent risk factor for CDI in patients in Cape Town, South Africa, and there has been at least one report of a CDI outbreak occurring in a tuberculosis hospital in the Eastern Cape province,^23^ suggesting the need for further research in this area.

While the overall burden of CDI in sub-Saharan Africa is largely unknown, data from the limited number of available studies suggest that the prevalence of CDI in the region is comparable to that of high income countries in Europe and North America.^24,25^ In South Africa, studies carried out in the Vhembe district of Limpopo province and Cape Town in the Western Cape report a prevalence of toxigenic *C. difficile* of between 10% and 20% in patients with diarrhoea.^26,27,28^ However, at least two of the previous studies undertaken in the country relied on diagnostic testing that detected toxin A alone, meaning that the rate of CDI in the country may be underestimated.^27,29^ Additionally, epidemiological information regarding CDI and the strains responsible for disease is currently lacking, particularly for HIV-positive/tuberculosis-positive patients, who may be at an increased risk of developing CDI. Therefore, the aim of the current study was to examine CDI in patients attending two specialist tuberculosis hospitals in Cape Town and to determine the molecular epidemiology of strains isolated from these patients.

## Methods

### Ethical considerations

All procedures performed in studies involving human participants were in accordance with the ethical standards of the institutional and national research committees and with the Helsinki Declaration and its later amendments. This study was approved by the Ethics Committee of the University of Cape Town (HREC Number: 310/2008). For this type of observational and retrospective study formal consent is not required.

### Study setting and sample collection

Adult patients with tuberculosis attending two specialist tuberculosis hospitals (designated Hospital A and Hospital B) in Cape Town between September 2014 and September 2015 were included as part of a larger surveillance study for CDI that has been published more fully elsewhere.^30^ Both hospitals house patients undergoing tuberculosis therapy with adult wards divided into long-stay multi-drug resistant (MDR) and extensively drug resistant (XDR) wards and short-stay separate male and female wards for drug sensitive patients. Inclusion criteria were clinical suspicion of CDI (based on a *C. difficile* diagnostic test request from the attending clinician) and an unformed stool sample, collected in sterile specimen containers. During the study period, stool samples submitted to the National Health Laboratory Service diagnostic laboratory at Groote Schuur Hospital that tested positive for *C. difficile* using the Xpert^®^ *C. difficile* platform (Cepheid, Sunnyvale, California, United States) were retained at −70 °C for further culture analysis. Recorded patient data were limited to patient identification number (necessary to identify repeat specimens), age, gender as well as the hospital and ward the patient was in at the time of sample submission.

### *C. difficile* isolation and characterisation

*C. difficile* was isolated from stool samples using cycloserine cefoxitin egg yolk agar.^31^ Briefly, stool was homogenised in phosphate buffered saline and heat shocked at 60 °C for 10 minutes, before being inoculated onto cycloserine cefoxitin egg yolk agar. Cultures were incubated for 24–48 hours at 37 °C in an anaerobic chamber under an atmosphere of 5% H_2_, 10% CO_2_ and 85% N_2_ (Forma Scientific, Model 1024, Marietta, Ohio, United States). Putative *C. difficile* isolates were confirmed by PCR targeting the *C. difficile tpi* gene, as well as the various toxin-encoding genes (*tcdA, tcdB, cdtA, cdtB*).^32,33^ Isolates were typed by capillary gel electrophoresis-based ribotyping^34^ and matched against a local strain database, which included a selection of profiles from the Cardiff Anaerobe Reference Unit (named according to the standard nomenclature) and the Swedish Institute for Communicable Disease Control (names preceded by ‘SE’ in the designation).^30^ Further characterisation by multilocus variable-number tandem-repeat analysis (MLVA) was carried out as previously described.^35^ Briefly, the calculated numbers of repeats at each locus were used to calculate a dissimilarity matrix based on the Manhattan distance between isolates. This dissimilarity matrix was used to construct minimum spanning trees using Kruskal’s algorithm implemented in the MSTgold program (version 2.4)^36^ with a final consensus tree generated from 2000 bootstrap replicates. Final tree editing was carried out using the Gephi graph visualiser (version 0.9.2).^37^ Strains with a total summed tandem-repeat difference of two or less across all loci were considered clonally related based on approaches used in previous studies.^35,38,39^

### Antibiotic susceptibility testing

Minimum inhibitory concentrations (MICs) for various antibiotics were determined using the gradient diffusion strip method. Testing was carried out using either ETEST^®^ strips (bioMérieux, Johannesburg, South Africa) (metronidazole, vancomycin, moxifloxacin and erythromycin) or MIC test strips (Liofilchem, Roseto degli Abruzzi, Italy) (rifampicin) and isolates were cultured on Brucella agar (supplemented with 5% horse blood, 5 *μ*g/ml haemin and 10 *μ*g/mL vitamin K). Clinical breakpoints were obtained from Clinical & Laboratory Standards Institute tables^40^ (metronidazole, moxifloxacin, erythromycin), from European Committee on Antimicrobial Susceptibility Testing tables^41^ (vancomycin), and published data (rifampicin).^42^ *C. difficile* ATCC 700057 and *C. difficile* 11/11 were used as control strains showing full susceptibility and reduced susceptibility to metronidazole respectively.^43^

## Results

### Xpert data

Between September 2014 and September 2015, initially, a total of 212 *C. difficile* test requests were received from the two hospitals. Slightly more than half (120; 56.6%) of the submitted samples were from female patients. Repeat specimens were obtained from a total of seven patients. Two of these were classified as duplicate specimens (received from the same patient within a two-week period) and were removed from subsequent analyses, while the remaining five were classified as recurrent disease, defined as including both relapse and reinfection cases. The minimum, maximum and median durations between tests for patients with recurrent disease were 60, 161 and 90 days, respectively. Samples were submitted from patients housed in nine different wards across the two hospitals. A total of 117 test requests (55.7%) were received from patients in drug sensitive (DS) tuberculosis wards, 77 (36.7%) from patients in MDR tuberculosis wards and four (1.9%) from patients in pre-XDR tuberculosis and XDR tuberculosis wards. A further 12 samples (5.7%) did not have ward information.

**FIGURE 1:**
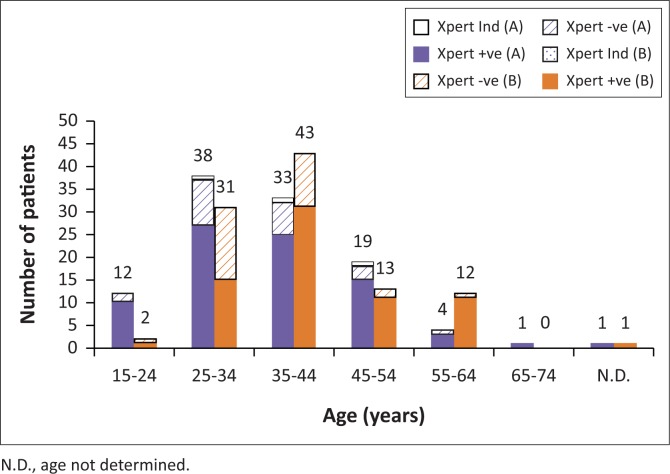
Detection of *C. difficile* in samples provided by patients attending Hospital A and Hospital B stratified by patient age. The relative proportions of samples provided by patients in Hospital A and Hospital B testing positive (+ve) and negative (-ve) by the Xpert^®^ *C. difficile* test (Xpert), along with those that yielded an indeterminate or invalid result (Ind). Numbers above the columns represent total number of tests performed for each category.

Overall, Xpert *C. difficile*-positive results were obtained for 152 samples (72.4%) in the non-repeat dataset (Figure 1). A total of three samples (1.4%) yielded indeterminate results and were not repeated. The period prevalence for Hospital A was approximately 84.05 cases per 1000 patient admissions and for Hospital B was approximately 60.03 cases per 1000 admissions (70.07 cases per 1000 admissions overall). Most samples (145; 69%) were submitted by patients between the ages of 24 and 45 and the median age for Xpert *C. difficile* positive patients (median 38, interquartile range 31–45.25) was slightly higher than for Xpert *C. difficile* negative patients (median 34, interquartile range 30–40).

### Frequency of *Clostridium difficile* infection cases stratified by ward

**FIGURE 2:**
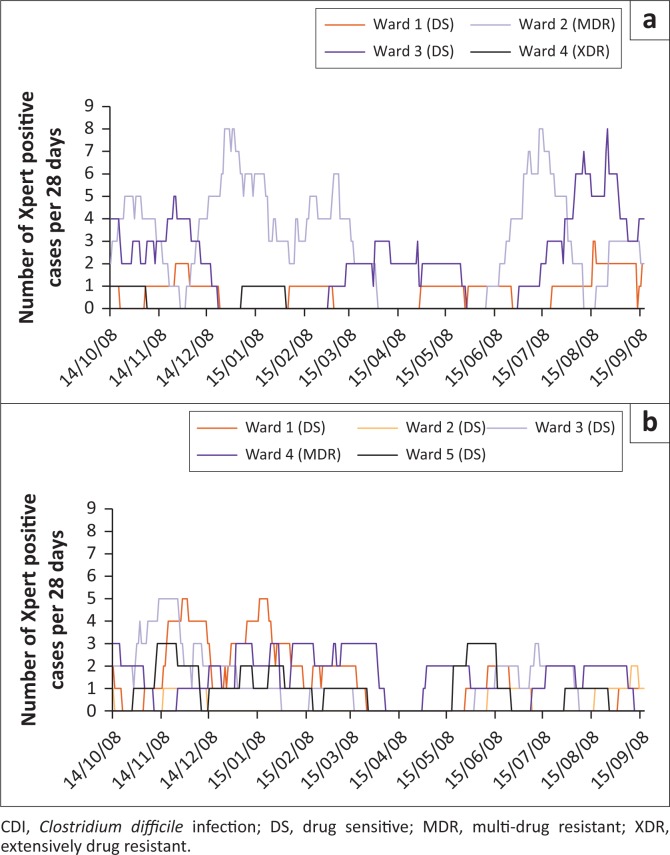
Frequency of CDI cases per ward. The number of CDI cases in 28-day windows for each ward in Hospital A (a) and Hospital B (b) over the study period. Windows move along the *x*-axis in one-day steps. Wards are designated as housing patients undergoing standard tuberculosis treatment (DS) or MDR/XDR treatment regimens (MDR/XDR).

The cumulative frequency of Xpert *C. difficile*-positive samples received from each ward in the two hospitals was analysed in 28-day windows for the entire study period, to allow the identification of periods of increased CDI prevalence (Figure 2). Hospital A experienced several periods of increased incidence (five or more samples per 28-day window). Ward 2 (MDR tuberculosis) showed four peaks, occurring throughout the study period. Ward 3 (DS tuberculosis) showed two peaks, the first of which occurred during the November–December 2014 period with the second occurring near the end of the study. Hospital B experienced three periods of increased incidence across two wards. Both ward 1 (DS tuberculosis) and ward 3 (DS tuberculosis) showed peaks during the November–December 2014 period, with an additional peak during January 2015 observed for ward 1.

### Isolation and molecular epidemiology of *C. difficile* strains

Of the 152 Xpert *C. difficile* positive samples, only 119 (78.3%) were retained for culture analysis mostly due to insufficient left over sample. Toxigenic *C. difficile* (*n* = 110) were isolated from 110 of these samples (92.4%). RT017, toxin A-B+ isolates accounted for 105 (95.5%) of the total number of strains isolated from patients in both DS tuberculosis and MDR tuberculosis wards. The remaining five toxin A+B+ isolates were typed as RT002 (one isolate), RT046 (one isolate) and RT(SE)108 (three isolates) and were all isolated from samples submitted by patients in DS tuberculosis wards from Hospital B.

**FIGURE 3:**
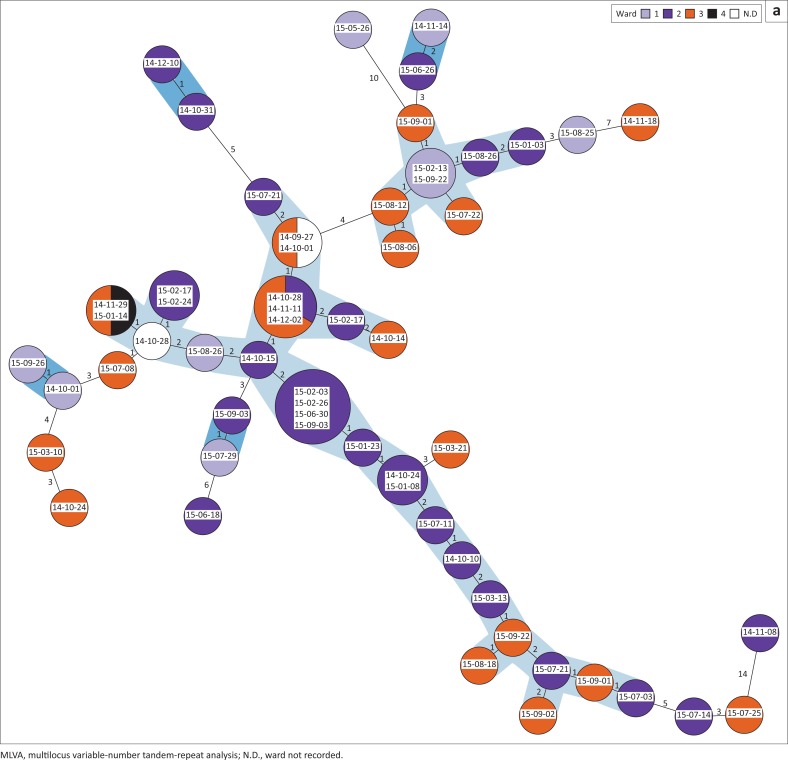

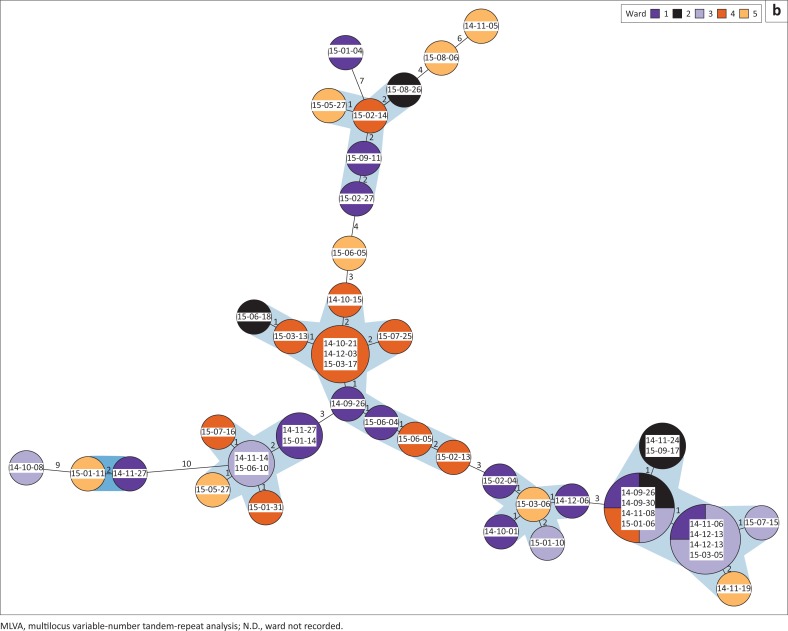
Minimum spanning tree of MLVA data for *C. difficile* RT017 isolates showing their clonal relationships and isolation sites. Strains isolated from Hospital A (a) and Hospital B (b) are represented by circles with the date of isolation included in the circle and the circle colour representing the ward that the patient was in at the time of sample submission. Larger circles represent multiple identical isolates, with the size of the circle proportional to the number of isolates. The total summed tandem-repeat difference between strains is given by the numbers between each circle. Clonally related isolates (summed tandem-repeat difference ≤ 2) are grouped within the shaded areas – () pairs, () clusters. The trees have been redrawn for ease of viewing and are not to scale.

MLVA revealed close relationships between many of the RT017 strains from the two hospitals. Just over half (53.4%) of all isolates from Hospital A clustered in one large group of clonally related strains (summed tandem-repeat difference ≤ 2) in a minimum spanning tree (Figure 3a). Strains in this group were isolated throughout the course of the study from patients in all four wards that experienced cases of CDI. A second smaller group of eight strains was also evident and comprised of strains isolated from three of the four wards between January 2015 and November 2015. Hospital B showed several groups of three or more clonally related strains (Figure 3b), which together accounted for 76.9% of all isolates. As for Hospital A, the clusters contained strains that were isolated from patients in different wards throughout the study period. It was noted that, in both hospitals, sets of three or four clonally identical strains were isolated from different patients in a single ward at different time points, suggesting possible patient-to-patient transfer events.

### Antibiotic susceptibility of isolates

Complete antibiotic susceptibility testing data (metronidazole, vancomycin, erythromycin and moxifloxacin) were available for a total of 77 isolates, 43 from patients attending Hospital A and 34 from patients attending Hospital B. Additional antibiotic susceptibility testing data were available for some strains and these were included in the analysis. All isolates from both hospitals were susceptible to vancomycin *in vitro* (Table 1). Similarly, none of the isolates was resistant to metronidazole, although four isolates from Hospital A showed reduced susceptibility to the antibiotic (MIC > 2 mg/L). All but one of the tested isolates (a RT002 isolate) were resistant to rifampicin. All tested RT017 isolates (n=72) were resistant to moxifloxacin, while the remaining non-RT017 isolates were susceptible to the antibiotic. Erythromycin resistance was observed in over two-thirds of the isolates (72.6%). Multi-drug resistance was observed for all tested RT017 strains (Table 2), with 73.6% of isolates resistant to erythromycin, moxifloxacin and rifampicin and a further 26.4% of isolates co-resistant to moxifloxacin and rifampicin. Co-resistance was also observed for RT(SE)108 strains, with two isolates resistant to erythromycin and rifampicin. There were no significant differences in antibiotic resistance patterns between wards and between hospitals.

**TABLE 1:** Antibiotic susceptibility data for isolates.

Antibiotic	MIC range (mg/L)	MIC_50_ (mg/L)	MIC_90_ (mg/L)	Susceptible		Intermediately resistant (%)	Resistant	
				%	Breakpoint mg/L		%	breakpoint mg/L
Vancomycin (*n* = 78)	0.25–1	0.5	0.75	100.00	≤ 2	0.00	0.00	≥ 8
Metronidazole (*n* = 97)	0.047–4	0.38	1.5	95.88	≤ 2	4.12	0.00	≥ 32
Erythromycin (*n* = 84)	0.38– > 256	> 256.00	> 256.00	25.00	≤ 2	2.38	72.62	≥ 8
Moxifloxacin (*n* = 87)	0.75– > 32	> 32.00	> 32.00	5.75	≤ 2	0.00	94.25	≥ 8
Rifampicin (*n* = 77)	< 0.016– > 256	> 256.00	> 256.00	1.30	≤ 0.016	0.00	98.70	≥ 16

*MIC*_*50*_, Minimum inhibitory concentration of 50% of isolates; *MIC*_*90*_, minimum inhibitory concentration of 90% of isolates.

**TABLE 2:** Multi-drug resistance by ribotype.

Ribotype	Sensitive to all antibiotics	Resistant to ERM or MXF or RIF only	Co-resistance (%)			
			ERM+MXF only	ERM+RIF only	MXF+RIF only	ERM+MXF+RIF
RT002 (*n* = 1)	1 (100)	0 (0)	0 (0)	0 (0)	0 (0)	0 (0)
RT017 (*n* = 72)	0 (0)	0 (0)	0 (0)	0 (0)	19 (26.4)	53 (73.6)
RT046 (*n* = 1)	0 (0)	1 (100)	0 (0)	0 (0)	0 (0)	0 (0)
RT(SE)108 (*n* = 3)	0 (0)	1 (33.3)	0 (0)	2 (66.7)	0 (0)	0 (0)

ERM, erythromycin; MXF, moxifloxacin; RIF, rifampicin.

## Discussion

There is a clear need for further study and increased surveillance of CDI in sub-Saharan Africa. Both hospital and community-acquired CDI cases have the potential to pose significant challenges to healthcare systems and there are currently no data on the economic burden of CDI outbreaks in the region. An inclusive ‘One-Health’ approach involving researchers at multiple levels in the community and hospital environment is important. A first step in this direction is the establishment of systematic sentinel surveillance of CDI in high-risk populations to monitor the incidence of CDI, as well as the predominant strain types and circulating antibiotic resistance phenotypes. Similar programmes in high income nations have been successful in reducing both the incidence of infection and mortality due to CDI^44^ and would be of benefit to countries in sub-Saharan Africa.

In the current study, CDI cases were examined in at-risk populations of patients undergoing long-term anti-tuberculosis therapy. CDI was common at both tuberculosis institutions with at least four wards experiencing spikes in the infection rates during the study period. Three of these wards housed patients undergoing DS tuberculosis treatment regimens, while one housed patients receiving MDR tuberculosis therapy. Although *C. difficile* outbreak definitions vary across countries, a rate of five or more cases in a single ward with at least 20 beds over a four-week period is generally used as a threshold to initiate outbreak investigations. That this occurred in four different wards during the study is concerning and suggests the need for enhanced surveillance of *C. difficile* in these hospitals to help to prevent further infections.

Toxin A-negative, toxin B-positive RT017 strains made up the majority of tuberculosis patient isolates. Strains belonging to this ribotype have been implicated in outbreaks in Canada,^45^ China,^46^ Korea,^47^ Argentina,^48^ Israel,^49^ Japan^50^ and Europe^6,51^ and they are often resistant to multiple drugs.^42,52^ Moreover, in previous studies RT017 strains have been shown to have a similar 30-day mortality rate of the so-called ‘hypervirulent’ RT027 strains.^5,53^ RT017 strains were also commonly isolated from other hospitals in Cape Town during the same time period, suggesting that these strains are circulating more broadly in the Cape Town patient population.^30,54^ It is possible that the previous use of standalone enzyme immunoassay diagnostic tests that target toxin A alone allowed RT017 strains to proliferate undetected in the region, providing a reservoir of strains in the healthcare environment with the potential to cause outbreaks in susceptible patient populations.

Several RT017 strains isolated from both hospitals were highly related by MLVA, suggesting possible patient-to-patient strain transfer. In a large scale epidemiological analysis of *C. difficile* strains infecting patients in England, Eyre et al.^55^ defined several possible transmission relationships for patients with clonally related *C. difficile* strains. For hospital-acquired cases, these included ‘ward contacts’ (two or more patients occupying the same ward over a concurrent time interval that allowed for direct patient-to-patient transmission), ‘ward contamination’ (patients occupying the same ward but with an interval of 1–28 days separating the discharge or end of infectivity of the initial case and the admission of patients who subsequently developed CDI) and ‘hospital contacts’ (patients in different wards at the same hospital over a similar time interval to ward contacts). Unfortunately, detailed ward occupation data and the duration of disease symptoms were not available for patients in this study and it is, therefore, not possible to differentiate between the three different transmission relationships in the current analysis. Nevertheless, the isolation of several clonally related strains from samples submitted by patients in the same ward within a 28-day time interval suggests that ward contact or ward contamination occurred along with possible hospital contact for samples from patients occupying different wards. Interestingly, strains with identical MLVA profiles were also isolated from different patients at longer time intervals of three to seven months. This has been observed previously^55^ and may be due to the formation of spores that persist in a genetically quiescent state on surfaces in the hospital environment.

Metronidazole is the recommended antibiotic for initial episodes of CDI, with vancomycin reserved for cases that do not respond to initial treatment and for cases of recurrent disease. No resistance to either of these antibiotics was observed for local isolates. However, a small number of strains exhibited a slightly elevated metronidazole MIC of 4 mg/L. This may be clinically relevant as the maximum level of metronidazole that can be maintained in the gut during oral therapy ranges between 0.25 mg/L and 9.5 mg/L.^1^ Fidaxomicin, an alternative treatment for CDI with a narrow spectrum of activity, is not yet widely available for use in South Africa.

Apart from one non-RT017 isolate, all *C. difficile* strains isolated from patients attending specialist tuberculosis hospitals were resistant to rifampicin. This included samples from both DS tuberculosis and MDR tuberculosis patients and contrasts with a concurrent set of isolates obtained from patients attending other hospitals in the Cape Town area (37% of strains resistant to rifampicin).^30^ It is also higher than that observed for strains isolated from the general hospital population in Europe (0% – 63.64%).^42^ Rifampicin is included as part of the standard DS tuberculosis treatment regimen and exposure to antibiotics belonging to this class has been associated with the development of resistance in *C. difficile*.^56^ Similar results have been observed for RT046 strains isolated from a tuberculosis hospital population in Poland.^16^ Resistance to rifamycin in *C. difficile* is usually associated with mutations in the *rpoB* gene, which typically confer cross-resistance to multiple members of the antibiotic class and, importantly, are associated with a low fitness burden, allowing the phenotype to be stably maintained in the bacterial population.^57^

Fluoroquinolone resistance was also common among *C. difficile* strains isolated from tuberculosis patients. Previous exposure to fluoroquinolones is a recognised risk factor for the development of CDI, and it is thought that acquisition of resistance to third and fourth generation fluoroquinolones by RT027 strains was a contributing factor to several hospital outbreaks in North America during the early 2000s.^4,58^ In South Africa, moxifloxacin is included as part of the MDR tuberculosis treatment regimen, and all *C. difficile* strains isolated from MDR tuberculosis patients were resistant to the antibiotic. Resistance to fluoroquinolones in *C. difficile* is chromosomally encoded,^59^ readily develops following exposure to this class of antibiotics^60^ and is associated with a low fitness cost to the bacterium.^61^ Therefore, the development of resistance to fluoroquinolones, as well as rifampicin, is likely to be stably maintained in the *C. difficile* population circulating among tuberculosis patients.

One reason proposed for the relatively low rates of CDI in patients undergoing anti-tuberculosis therapy in previous studies is that rifampicin is often effective against *C. difficile* and may help to protect against CDI in these patients.^20^ Since this protection would be lacking in a background of circulating rifampicin-resistant strains, the presence of rifampicin resistance among the RT017 isolates in the current study is significant. Moreover, co-resistance to fourth generation fluoroquinolones would allow the same strains to persist in patients undergoing MDR tuberculosis treatment regimens. Together, these results may help to explain the increased risk of CDI in tuberculosis patients in Cape Town^22^ compared to previous studies in Hong Kong^20^ and Korea^21^ and confirm the need to monitor this patient population more carefully.

### Limitations

There are several limitations to the current study. As mentioned, detailed ward occupation data were not available, and this will need to be collected in future analyses to identify various potential strain transmission routes. Additionally, while MLVA has previously been shown to be as sensitive as whole genome sequencing in tracking *C. difficile* transmission,^38^ it has been noted that RT017 strains show very little variation for several of the loci included in the scheme,^30,35^ suggesting that the method may not be suitable for examining RT017 transmission dynamics. Therefore, we are currently performing whole genome sequencing on selected isolates to determine the fine-scale molecular epidemiology of local RT017 strains in order to complement the MLVA results.

### Conclusion

The presence of a relatively large number of MDR RT017 *C. difficile* strains in patients attending specialist tuberculosis hospitals in Cape Town is noteworthy, especially given their potential to cause outbreaks. Many of the isolates were closely related by MLVA, and there is some evidence to suggest that patient-to-patient transfer of strains took place during the study period. Tuberculosis patients, many of whom are co-infected with HIV, may represent a population that is vulnerable to CDI and further studies should be undertaken to evaluate the risk in this patient group. Additionally, the propensity of *C. difficile* to form highly resistant spores that are continually shed by individuals with CDI warrants additional studies to investigate potential contamination of surfaces present in the hospital environment. Tuberculosis patients who do not go on to develop active CDI may still be colonised and subsequently become carriers of the organism to the community or other healthcare facilities. Finally, information regarding *C. difficile* in other sub-Saharan countries is currently lacking and further research is needed to understand the epidemiology of the organism in the region more thoroughly.
